# Supplementary material for: Assembly of a G-Quadruplex Repair Complex by the FANCJ DNA Helicase and the REV1 Polymerase
Source: Genes (Basel). 2019 Dec 19;11(1):5. doi: 10.3390/genes11010005 (PMC7017153; doi:10.3390/genes11010005)
Supplement: Supplementary file 1 [file genes-11-00005-s001.zip › Supplementary Materials 121819.docx]

**Supplementary Materials**

**Assembly of a G-quadruplex Repair Complex by the FANCJ DNA Helicase and the REV1 Polymerase**

Kaitlin Lowran^1^, Laura Campbell^1^, Phillip Popp^2^, and Colin G. Wu^1,^*

**Figure S1. CD spectra of (TTAGGG)_4_ and (GGGT)_4_ in Buffer H with 150 mM NaCl.** (**A**) The human telomeric G4 DNA adopted an anti-parallel configuration in NaCl buffer. 8-oxoguanine modifications at the 8oxo1 or 8oxo5 positions had little effect on G4-folding. (**B**) (GGGT)_4_ substrate with or without DNA damage retained its parallel conformation in NaCl buffer.

**Figure S2. BLI time-courses of the FANCJ AKKQ peptide binding to 8oxoG4s.** (**A**) Biotinylated human telomeric G4 DNA with an 8oxo1 was immobilized on streptavidin-coated biosensors. Freely diffusing FANCJ AKKQ peptide was introduced at 32, 16, 8, 4, 2, 1, and 0.5 µM to initiate the association reactions. The BLI biosensors were placed in Buffer H (red line) to examine the dissociation of the 8oxoG4-AKKQ complexes. (**B**) Similar experiments were performed with a biotinylated 8oxo5 substrate and 32, 16, 8, 6, 4, 3, 2, 1.5, 1, 0.75, and 0.5 µM FANCJ AKKQ. These trajectories were used to construct the binding isotherms shown in Figure 3C.


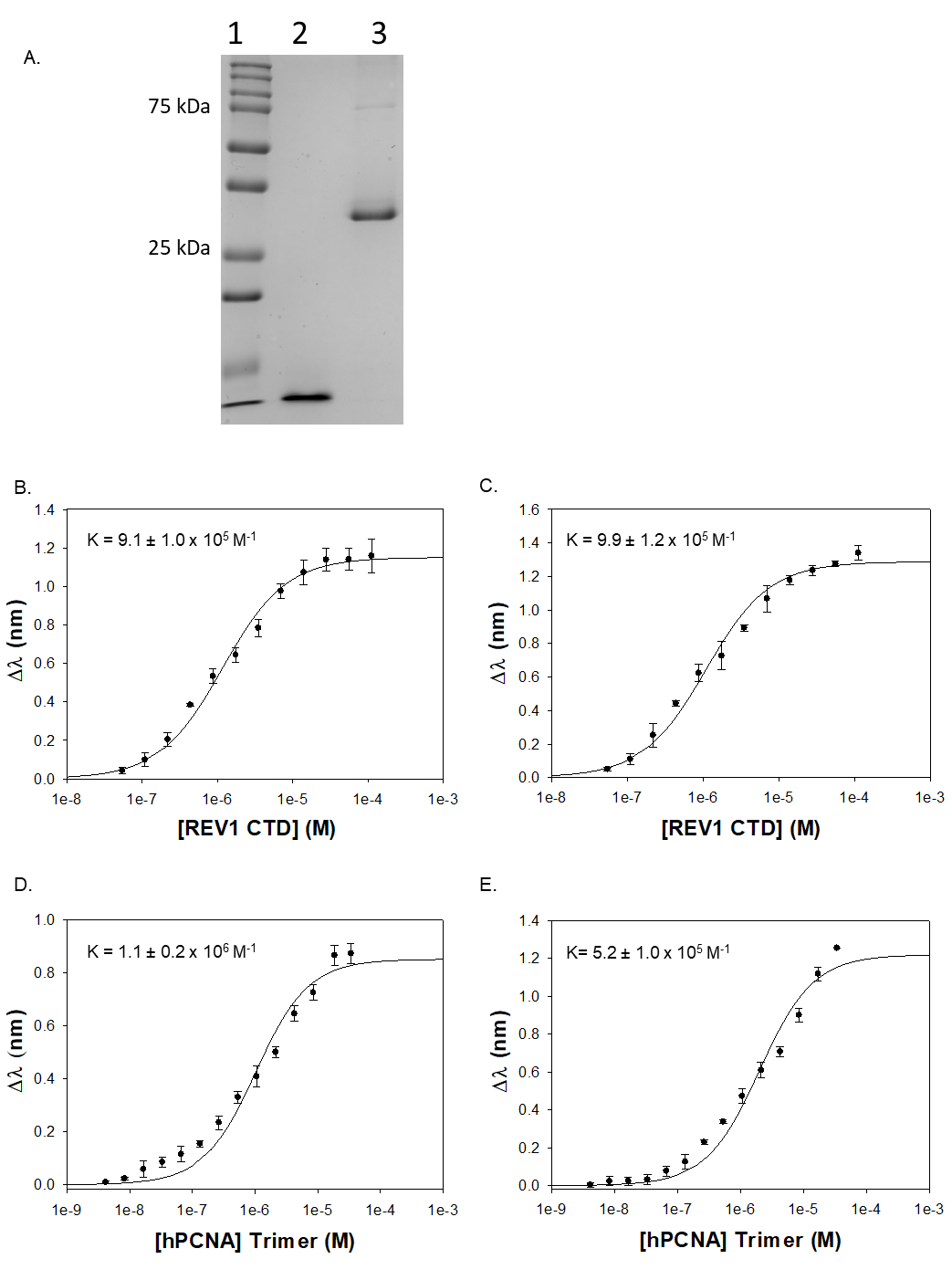


FANCJ PIP vs. REV1 CTD

FANCJ PIP AA vs. REV1 CTD

FANCJ PIP AA vs. PCNA

FANCJ PIP vs. PCNA

**Figure S3. BLI binding experiments with FANCJ PIP, REV1 CTD, and PCNA.** (**A**) SDS-PAGE of purified recombinant proteins. A ladder of known molecular sizes were separated in Lane 1. The 75 kDa and 25 kDa bands were marked as references. Purified full-length human PCNA was loaded in Lane 2. The PCNA monomer was resolved as a homogeneous band at ~30 kDa. Purified REV1 CTD was loaded in Lane 3. The protein migrated as a ~12 kDa band. (**B** and **C**) Binding isotherms of REV1 CTD interacting with wild-type FANCJ PIP (**panel B**) or the PIP AA mutant (**panel C**). (**D** and **E**) Isotherms of PCNA binding to wild-type FANCJ PIP (**panel D**) or the PIP AA mutant (**panel E**).
