# Supplementary figures and images for: Assembly of a G-Quadruplex Repair Complex by the FANCJ DNA Helicase and the REV1 Polymerase
Source: Genes (Basel). 2019 Dec 19;11(1):5. doi: 10.3390/genes11010005 (PMC7017153; doi:10.3390/genes11010005)

Figure S1

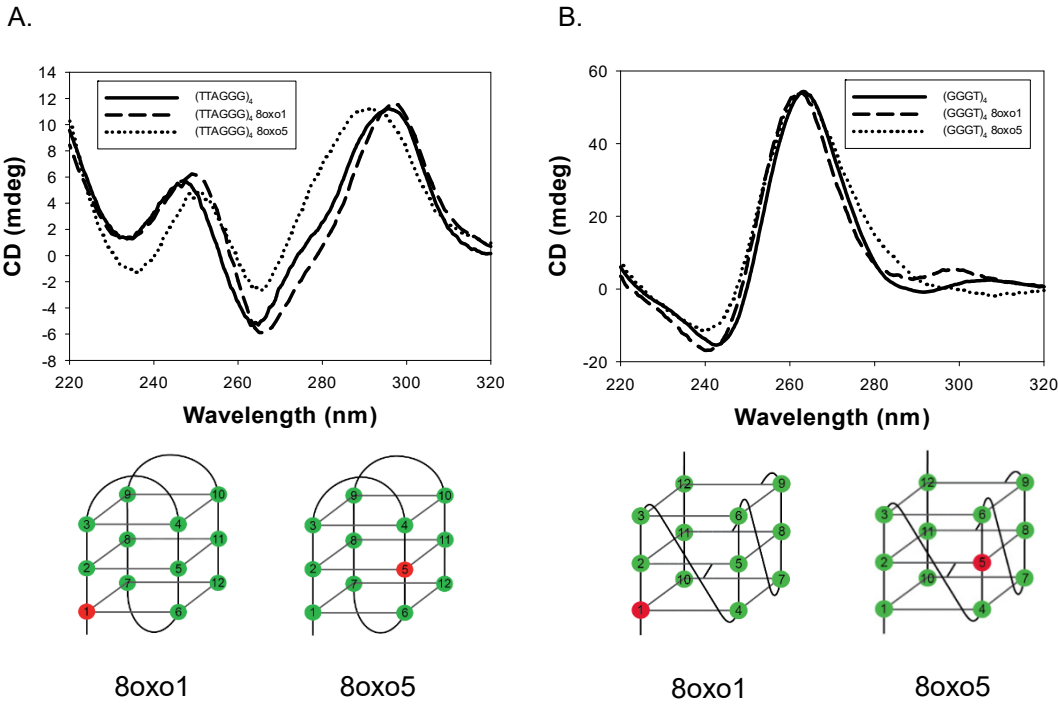

Figure S2

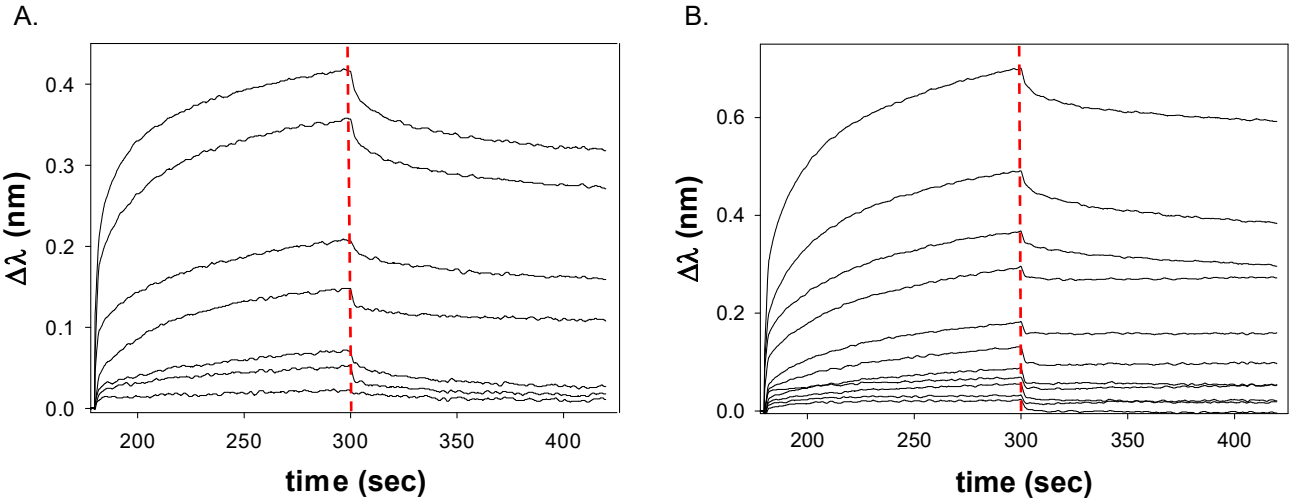

Figure S3

A.

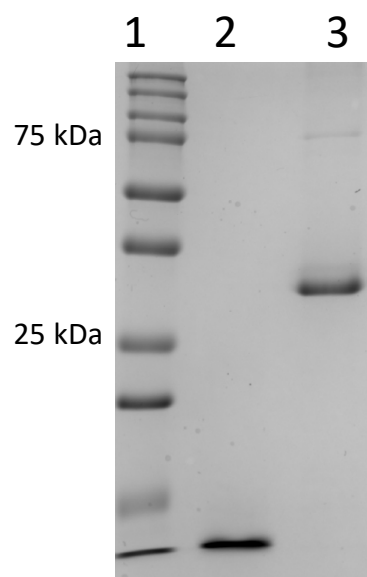

B.

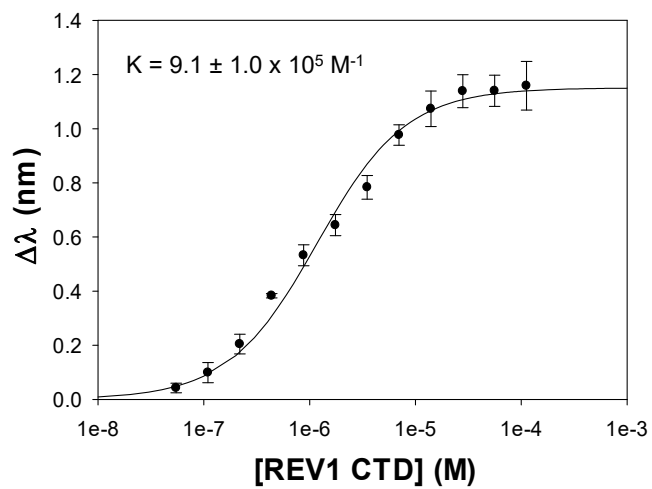

C.

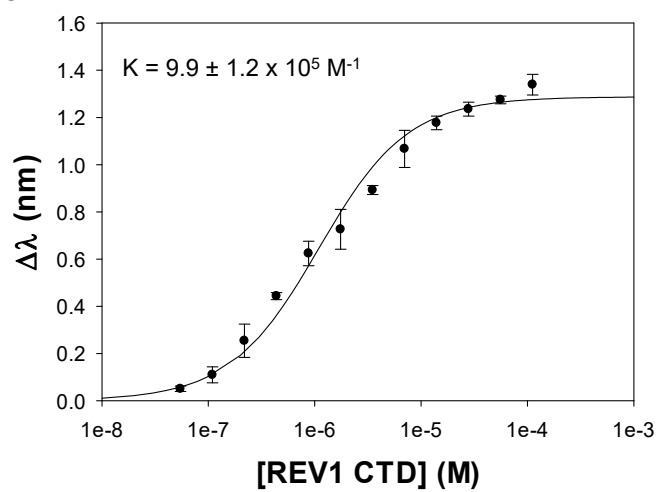

D.

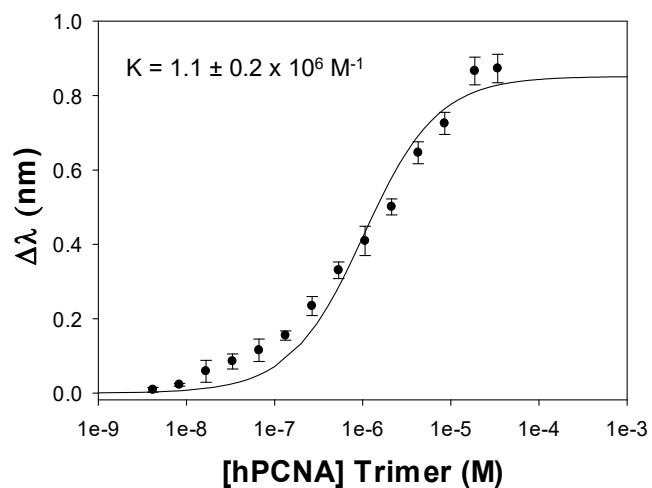

E.

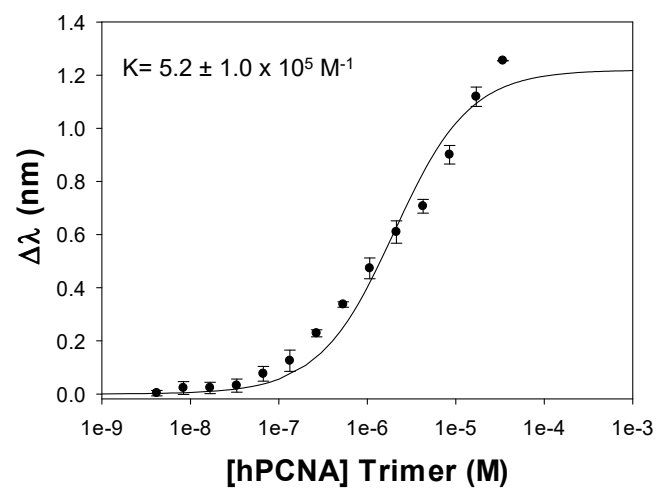

Supplement: Supplementary file 1 [file genes-11-00005-s001.zip › Supplementary Figures 121819.pdf]

## Slide 1
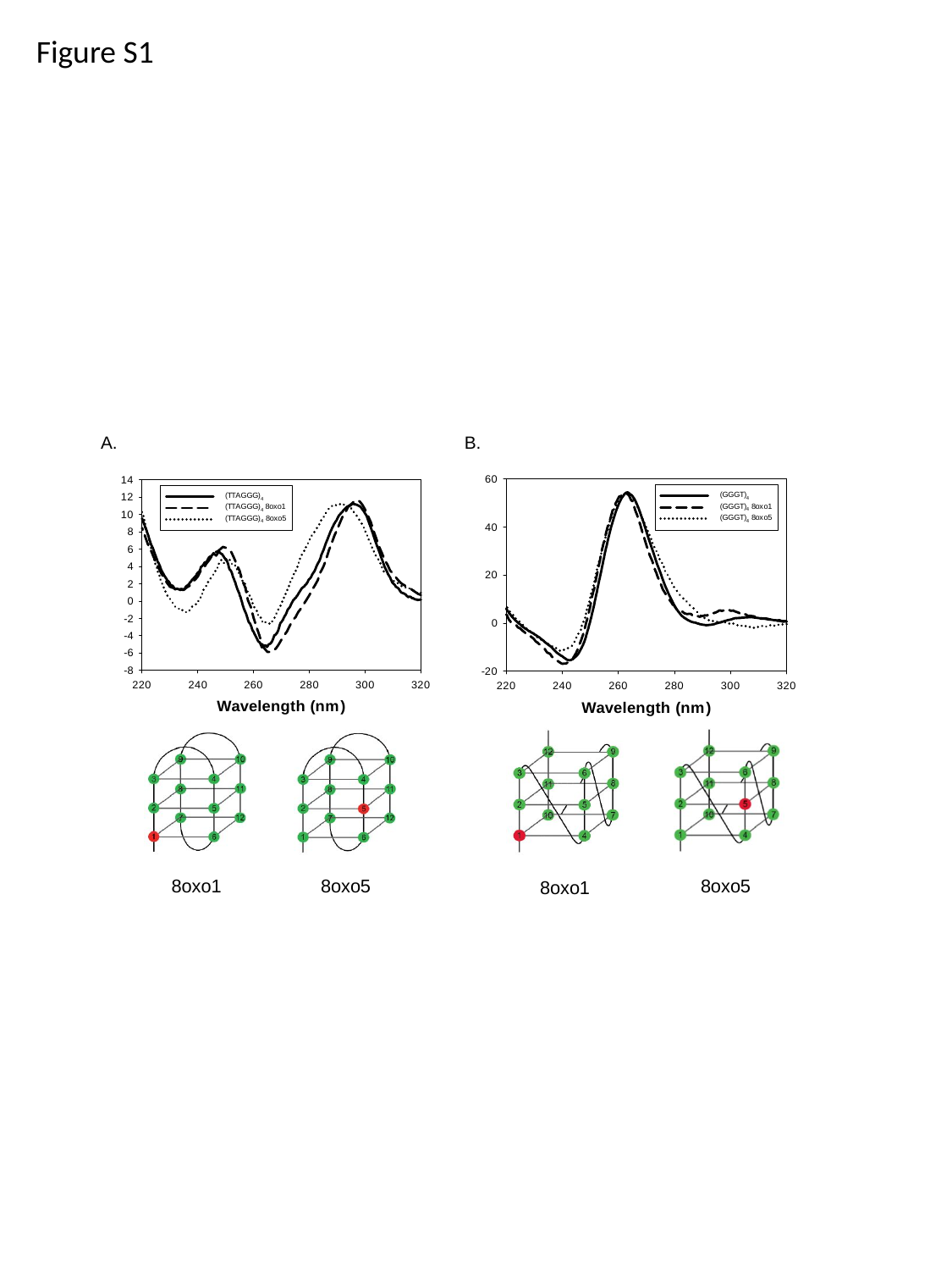

Figure S1
B.
A.
8oxo1
8oxo5
8oxo5
8oxo1

## Slide 2
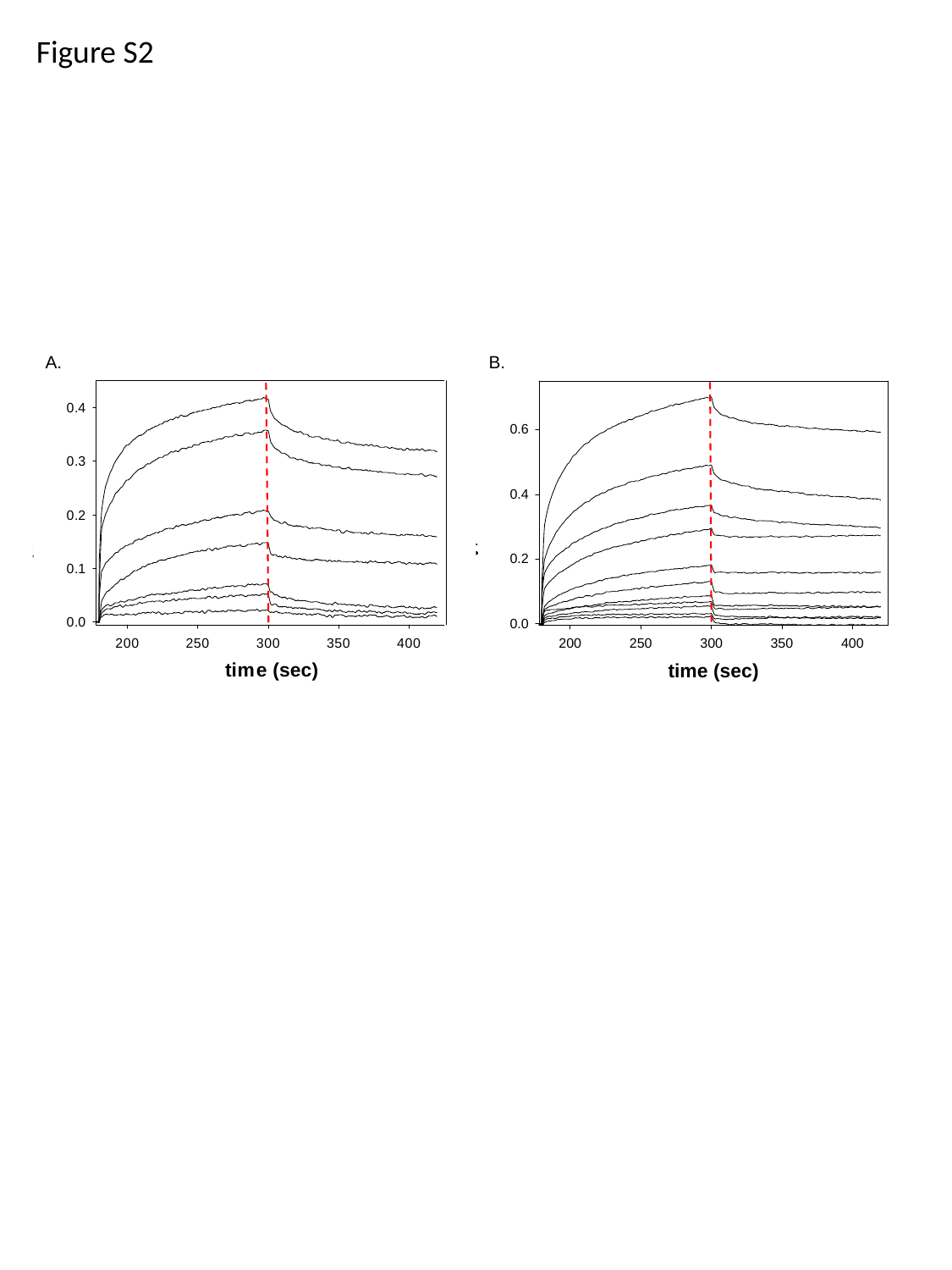

Figure S2
A.
B.

## Slide 3
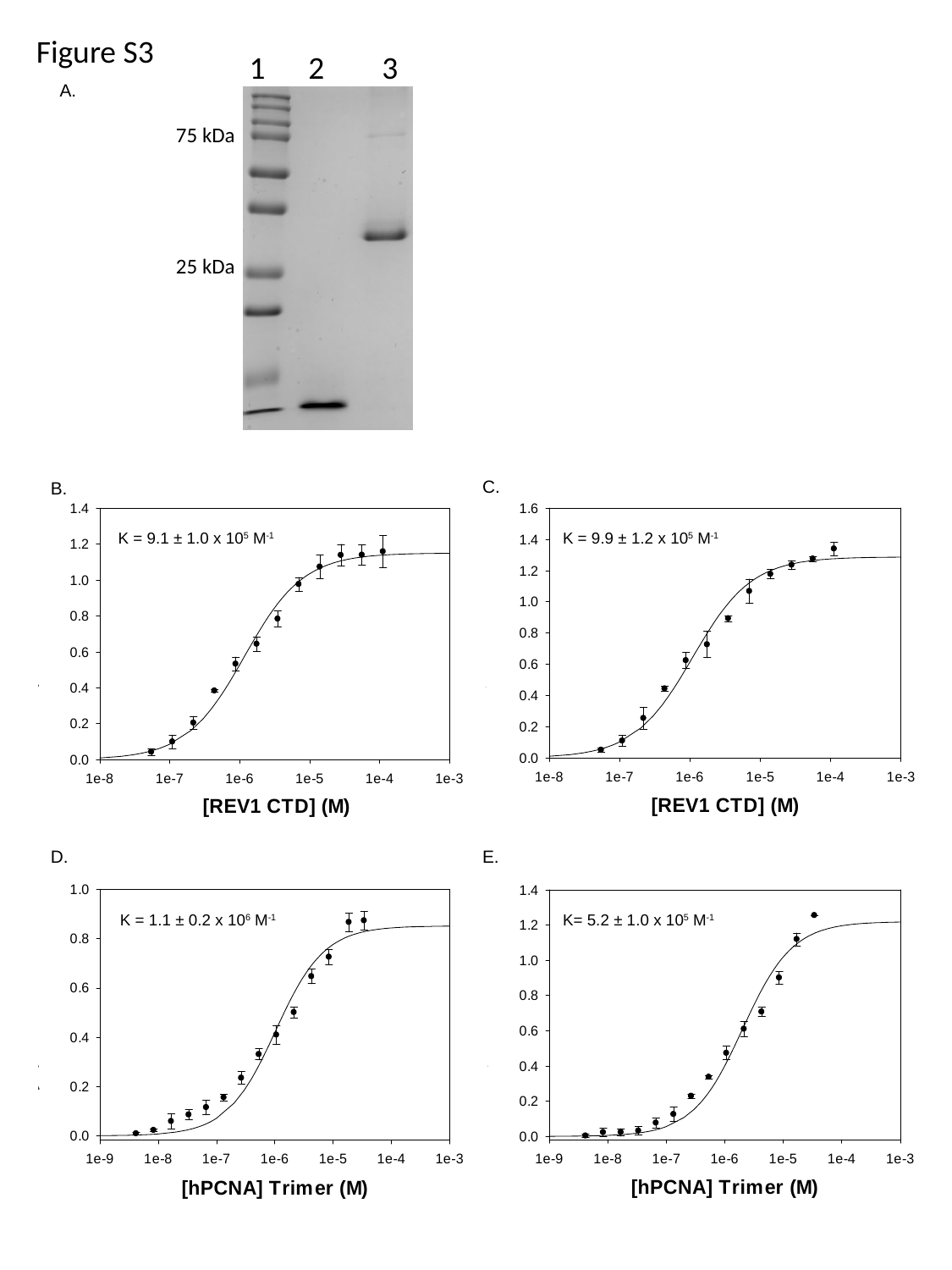

Figure S3
1 2 3
A.
75 kDa
25 kDa
C.
B.
K = 9.1 ± 1.0 x 105 M-1
K = 9.9 ± 1.2 x 105 M-1
D.
E.
K = 1.1 ± 0.2 x 106 M-1
K= 5.2 ± 1.0 x 105 M-1

Supplement: Supplementary file 1 [file genes-11-00005-s001.zip › Supplementary Figures 121819.pptx]
